# Comprehensive Molecular Profiling of NPM1-Mutated Acute Myeloid Leukemia Using RNAseq Approach

**DOI:** 10.3390/ijms25073631

**Published:** 2024-03-24

**Authors:** Jessica Petiti, Ymera Pignochino, Aurora Schiavon, Emilia Giugliano, Enrico Berrino, Giorgia Giordano, Federico Itri, Matteo Dragani, Daniela Cilloni, Marco Lo Iacono

**Affiliations:** 1 Division of Advanced Materials Metrology and Life Sciences, Istituto Nazionale di Ricerca Metrologica (INRiM), 10135 Turin, Italy; j.petiti@inrim.it; 2 Department of Clinical and Biological Sciences, University of Turin, 10043 Orbassano, Italy; ymera.pignochino@unito.it (Y.P.); aurora.schiavon@unito.it (A.S.); federico.itri@unito.it (F.I.); daniela.cilloni@unito.it (D.C.); 3 Candiolo Cancer Institute, FPO-IRCCS, 10060 Candiolo, Italy; enrico.berrino@unito.it (E.B.); giorgia.giordano@unito.it (G.G.); 4 Clinical and Microbiological Analysis Laboratory, San Luigi Gonzaga Hospital, 10043 Orbassano, Italy; e.giugliano@sanluigi.piemonte.it; 5 Department of Medical Sciences, University of Turin, 10126 Turin, Italy; 6 Department of Oncology, University of Turin, 10043 Orbassano, Italy; 7 Division of Hematology and Cellular Therapies, San Martino Hospital, IRCCS, 16132 Genova, Italy; matteoemidio.dragani@hsanmartino.it

**Keywords:** NPM1, next-generation sequencing, acute myeloid leukemia, RNA/DNA variant calling

## Abstract

Acute myeloid leukemia (AML) is a complex hematologic malignancy with high morbidity and mortality. Nucleophosmin 1 (NPM1) mutations occur in approximately 30% of AML cases, and NPM1-mutated AML is classified as a distinct entity. NPM1-mutated AML patients without additional genetic abnormalities have a favorable prognosis. Despite this, 30–50% of them experience relapse. This study aimed to investigate the potential of total RNAseq in improving the characterization of NPM1-mutated AML patients. We explored genetic variations independently of myeloid stratification, revealing a complex molecular scenario. We showed that total RNAseq enables the uncovering of different genetic alterations and clonal subtypes, allowing for a comprehensive evaluation of the real expression of exome transcripts in leukemic clones and the identification of aberrant fusion transcripts. This characterization may enhance understanding and guide improved treatment strategies for NPM1mut AML patients, contributing to better outcomes. Our findings underscore the complexity of NPM1-mutated AML, supporting the incorporation of advanced technologies for precise risk stratification and personalized therapeutic strategies. The study provides a foundation for future investigations into the clinical implications of identified genetic variations and highlights the importance of evolving diagnostic approaches in leukemia management.

## 1. Introduction

Acute myeloid leukemia (AML) is a complex and aggressive hematologic malignancy characterized by the uncontrolled proliferation and accumulation of immature myeloid cells in the bone marrow and peripheral blood. AML accounts for about 80% of adult leukemia cases and is associated with high morbidity and mortality, with a 5-year relative survival rate lower than 30% [1]. AML is a heterogeneous disease, characterized by several genetic variations (GV), such as gene mutations and cytogenetic abnormalities, defining numerous subcategories with distinct prognoses and therapies. Among the diverse molecular subtypes of AML, mutations in the nucleophosmin 1 (NPM1) gene have emerged as a distinctive and clinically significant genetic alteration. Indeed, NPM1 is one of the most commonly mutated genes, occurring in approximately 30% of adult leukemia cases with normal karyotype [2]. The NPM1 gene, located on chromosome 5q35, encodes for a multifunctional protein involved in various cellular processes, including ribosome biogenesis, centrosome duplication, and maintenance of genome stability [3]. In AML, NPM1 mutations frequently involve exon 12, rarely exon 6, 9, and 11, leading to aberrant cytoplasmic localization of the protein. This alteration results in disrupted nucleocytoplasmic shuttling, impaired protein–protein interactions, and dysregulated cell proliferation and survival pathways [2].

Due to its unique clinical and molecular features, the World Health Organization (WHO) Classification of Haemato-lymphoid Tumours [4] identifies the NPM1-mutated (mut) AML as a distinct entity irrespective of blast counts, known as “AML with mutated NPM1”. NPM1mut is frequently associated with a normal karyotype and can manifest independently or in conjunction with additional genetic variations affecting genes such as FLT3, DNMT3a, IDH1, IDH2, and TET2 [5]. According to the WHO Classification, NPM1mut AML patients without any other genetic abnormalities are associated with a more favorable prognosis compared to other AML subtypes [4]. Indeed, literature data described these patients as characterized by enhanced disease-free survival (DFS) and overall survival (OS), coupled with a diminished incidence of relapse [6]. Schneider et al. report that 75% of NPM1mut AML patients without any other genetic abnormality achieve complete remission after the first induction cycle. This highlights the remarkable in vivo susceptibility of NPM1mut cells to induction chemotherapy [7]. Despite these considerations, about 30–50% of NPM1mut AML patients relapse usually within 12–18 months after the first complete remission [8]. Relapse, with or without NPM1mut and other concomitant somatic mutations, can still happen for various reasons, such as subclonal heterogeneity, persistence of residual disease/therapeutic resistance, or genetic evolution. Subclonal heterogeneity in AML is frequent: while NPM1 mutations may drive the initial leukemic process and response to therapy, other subclonal mutations or genetic alterations, not detected at diagnosis, could contribute to relapse. These undetected mutations might confer resistance to treatment or enable the emergence of more aggressive cell populations. 

The presence of co-occurring mutations can affect the otherwise favorable prognosis of NPM1mut patients. For instance, the presence of an FLT3-ITD mutation changes the risk profile of a patient with mutated NPM1 from favorable to intermediate [9]. Although in 2022 the European LeukemiaNet (ELN) prognostication systems [9] highlighted the growing importance of molecular data obtainable from massively parallel sequencing, the adoption of NGS for routine diagnostics can vary widely among different healthcare institutions, regions, and countries. 

The experimental hypothesis driving this work was, therefore, based on the idea that somatic mutations, already present at diagnosis, can impact the outcome of these patients. To address this experimental question and implement new-generation technologies in the diagnosis of AML, we evaluated the use of RNAseq for more accurate molecular characterization of patients. To increase knowledge of NPM1mut AML, we evaluated the genetic variations (GVs) independently by a myeloid stratification. Our results suggest how the use of total RNA sequencing techniques can be suitable for the study of the leukemic patient, who exhibits a mosaic of peculiar low-frequency genetic variations and clonal subtypes. This approach may contribute to a more comprehensive and accurate characterization of NPM1mut AML, potentially improving treatment strategies and outcomes for patients.

## 2. Results

### 2.1. Patient Cohort

We carried out a retrospective study on diagnostic material extracted from AML patients with mutated NPM1, a normal karyotype, and defined at diagnosis as wild type for FLT3 and other myeloid-associated mutations. The cohort consisted of 10 males and 13 females with a mean age at diagnosis of 66.4 years, ranging from 30 to 86 years. Among the 23 patients evaluated, 9 (39%) experienced relapses, and 8 died (34%).

To identify somatic mutations that may have been present at diagnosis but were not identified by conventional diagnostic methods, potentially leading to misclassification of the risk of NPM1mut AML, we evaluated the use of NGS on both DNA and RNA diagnostic samples. This approach aimed to provide a more accurate molecular analysis for the comprehensive characterization of patients.

### 2.2. Targeted DNA Sequencing Analysis Revealed Additional Pathogenic Mutations

**Figure 1:**
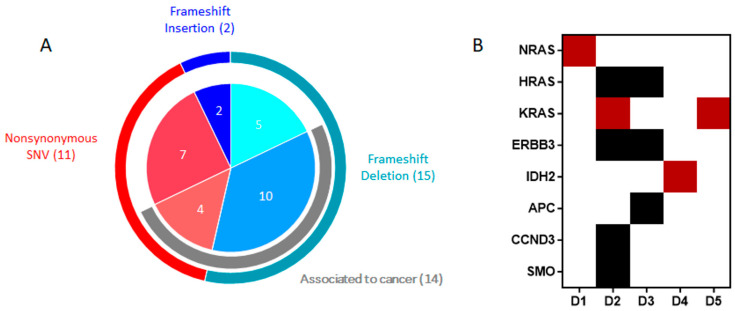
Genetic variations identified in targeted DNA sequencing analysis. (**A**) The outer ring identifies the GV subtypes, the gray arc highlights the GVs associated with cancer, while the inner pie chart identifies the total number of GVs found for each subgroup. (**B**) The heatmap shows the genes hit by non-synonymous variations for each patient identified by targeted DNA sequencing analysis. The garnet GVs already associated with hematological diseases are highlighted (dark red). D: DNA ID.

In Figure 1, we indicate all genetic variations identified through the application of filters for DNAseq, subdivided by the type of genetic variation. We identified a total of 28 genetic variations (sequencing depth > 100) in 13 out of 17 patients. Frameshift deletions exhibited the highest prevalence, followed by non-synonymous variations and frameshift insertions (Figure 1A). Although the identified genetic frameshift variations impact repeated genetic regions, which could potentially introduce false positive errors attributable to alignment and sequencing inaccuracies, we identified 17 frameshifts, of which 10 are cancer-associated in ClinVar. In particular, 5 were SNPs affecting the PTEN gene (rs786204900 (4 pts), rs121912595 (1 pt)) and 5 the TP53 gene (1 SNP: rs876660726 (3 pts) and 2 with COSMIC ID: COSM100028 (1 pt), COSM96581(1 pt)). Focusing on non-synonymous variations (Supplementary Table S1), we identified variations in the NRAS, HRAS, KRAS, ERBB3, IDH2, APC, CCND3, and SMO genes in 5 patients (Figure 1B). Intriguingly, 4 of these non-synonymous variations, found in 4 different patients, were previously associated in the literature with AML: NRAS p.G13R (NM_002524) identified in heterozygosis with an allelic frequency (AF) of 34%; KRAS p.Q61H (NM_001369786; AF = 6%); KRAS p.G13D (NM_001369786; AF = 12%); and IDH2 p.R140Q (NM_002168; AF = 6%).

### 2.3. Total RNA Sequencing Analysis Showed the Transcription of Mutated Genes

**Figure 2:**
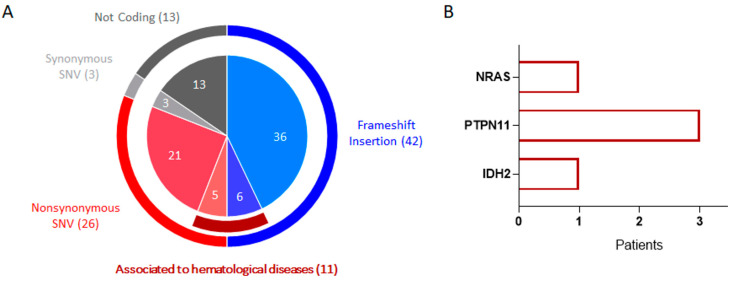
Genetic variations identified by total RNA sequencing analysis. (**A**) The outer ring identifies the GV subtypes already observed in tumors, while the inner pie chart shows the subgroups associated with hematological diseases, also highlighted by the garnet arc. (**B**) The bar graph shows the frequency of non-synonymous variations associated with hematological diseases identified in patients for each gene.

The RNAseq results provided a more complex scenario, showing the transcription of some of the mutations identified by targeted DNA sequencing analysis and complementing with many others. Using a filter of at least 100 sequenced fragments, we identified genetic variations already associated with cancer in the literature across all 18 patients evaluated: 42 frameshift insertions, 13 not associated with genetic function, 26 non-synonymous SNVs, and 3 synonymous SNVs (Figure 2 and Supplementary Table S2). By restricting the analysis exclusively to mutations observed in hematological diseases annotated in the ClinVar database, we found 11 genetic variations distributed among 10 patients: 6 frameshift insertions and 5 non-synonymous SNVs. Small insertion/deletion of NPM1 was found in 6 out of 18 cases. This stringent bioinformatic pipeline did not permit the identification of the confirmed NPM1 insertions/deletions present in some cases (Supplementary Figure S1). The results are summarized in Figure 2, where, among the GVs associated with hematological diseases, we identified: 3 patients with a mutation in exon 3 of PTPN11 gene (transcript NM_001330437: AA substitution p.A72V with a variant transcripts ratio (TR) of 31%; NM_001330437:p.E76K, TR = 48% and NM_001330437:p.A72T, TR = 24%); 1 patient with a mutation in exon 2 of IDH2 (NM_002168:p.R140Q, TR = 36%); and another patient with a mutation in exon 2 of NRAS (NM_002524:p.G12D, TR = 48%). 

Although a filter of at least 100 sequences could be useful for detecting mutations in the genome [10], such stringency might not be optimal for mutational analyses based on transcriptomics. In fact, the quantity of sequences in a certain area depends on the expression of that genetic locus, rather than the technical execution of sequencing. For this reason, we tried to broaden the filter to 25 transcripts. While this threshold was somewhat arbitrary, it may enable the identification of genetic variations in poorly expressed genes and/or in tumor subclones. The results of the modified filter are summarized in Table 1 and Supplementary Table S2. Intriguingly, in comparison to the previous analysis, we observed 6 new non-synonymous SNVs linked to hematological disease, including variations in CBL (1 pt); DNMT3A (3 pts); FLT3 (1 pts), NQO1 (1 pt); NRAS (1 pts) and 2 frameshifts: CEBPA (1 pts) and NBN (1 pts). Noteworthily, the genetic variation NRAS p.G13R was already identified in the same patient at the DNA level in the previously reported analysis, with an AF of 34%. 

### 2.4. Translocation Analysis

Another advantage of total RNAseq analysis is the possibility of identifying genetic alterations, such as gene fusion and the expression of non-canonical transcripts. Using STAR-Fusion, we identified some transcript alterations in 14 out of 18 patients. In most cases, we identified splicing variations that could create altered transcriptions. Among them, two intrachromosomal aberrations had a higher frequency in the analyzed patients. Within the group of rearrangements using the canonical splice site, RP11-367G6.3--FAM65B on chromosome 6 had the highest frequency and was observed in 7 patients, while the local rearrangement RP11-632K20.2--RP11-632K20.7 on chromosome 15 was present in 4 patients (pts) in our cohort. Notably, some non-canonical transcripts identified could interfere with the potential tumor suppressor SMG1 gene (2 pts) or with the oncogenes NDRG1 (1 pt) and SOX5 (1 pt). At the inter-chromosomal level, we observed translocations between chromosomes 22 and 14 that involved some intergenic regions and the locus of the HIRA gene (Table 2).

In Table 3, we summarize a comprehensive analysis for each patient. Our analysis identified a more complex scenario than the “standard” evaluation of patients with NPM1 mutations. Among the 23 patients analyzed with RNAseq or DNAseq, 10 showed at least one genetic variation associated with leukemia. This count increased to 17 if we also included transcript alterations in the analysis.

## 3. Discussion

The experimental hypothesis guiding this work stemmed from the potential failure in identifying somatic mutations already present at diagnosis in NPM1mut AML patients. This could lead to a misclassification of the risk associated with NPM1mut AML and, consequently, to ineffective treatment, followed by the relapse of the incorrectly characterized patient. To answer this experimental question and integrate advanced technologies into AML diagnosis, we explored the use of next-generation sequencing (NGS), including genetic variations identified by RNAseq analysis, for more accurate molecular characterization of patients.

Our analysis identified a more complex scenario than the conventional evaluation of patients with NPM1 mutations, as summarized in Table 3. The predominant mutations identified in our work affect the PTPN11 gene. In particular, we observed two mutations in the Src homology 2 domains (C-SH2), leading to the substitution of alanine in position 72 with valine or tryptophan, and another mutation substituting glutamic acid in position 76 with lysine (E76K). Notably, the E76K substitution is the most commonly identified somatic PTPN11 mutation in juvenile myelomonocytic leukemia. The E76A mutant displays elevated phosphatase activity, enhancing interleukin 3 (IL-3)–independent survival of transduced cells [11]. PTPN11 mutations are recurrent alterations in AML patients and serve as an independent prognostic factor for poor survival. Worse clinical outcomes have been observed in patients within the European LeukemiaNet favorable cytogenetics risk group [12]. In line with the literature, one of the patients in our cohort exhibited a co-occurrence of a PTPN11 mutation with a FLT3 mutation that had not been identified with conventional diagnostic technologies. Specifically, the FLT3 mutation identified in this case was at position D839, mapping to the activation loop of FLT3. This mutation stabilizes the conformation of FLT3 in the autoinhibited state, resulting in a more flexible activation loop and, consequently, a higher propensity for tyrosine phosphorylation. The presence of a mutation in the FLT3 gene changes the patient’s risk stratification, from favorable to intermediate. Furthermore, given that mutated FLT3 is recognized as a poor prognostic factor for long-term survival in AML patients, targeting FLT3 holds promise as an effective therapeutic strategy. Indeed, the development of FLT3 inhibitors represents a significant advancement in leukemia therapy, offering potential ways for improved treatment outcomes [13,14,15]. To mitigate the risk of relapses and/or the development of drug resistance, it is important to characterize the FTL3 mutational profile, allowing for accurate and individualized patient risk stratification based on specific mutations. Additionally, at the therapeutic level, also the early identification of mutations on IDH1 and 2 genes could potentially be important to correctly direct the patient to a more appropriate and targeted therapy. Mutations in IDH1 and IDH2 have been associated with distinct molecular and clinical features in AML. The most frequent mutations of IDH2, impacting over 95% of patients with IDH2 mutations, predominantly involve the arginine residues at positions R140 and R172 [16], while approximately 90% of IDH1 mutations in AML involve the substitution of arginine for histidine at position 132 (R132H) [17]. In our analysis, we identified two different mutations of IDH2 at the R140 position (R140Q), one at the DNA level in a patient without RNA specimens and the other at the RNA level. This latter mutation was also present at the DNA level in the original aligned BAM file. However, it was filtered out by the algorithm due to the abundance of variations in the surrounding area. In NPM1mut AML cases, the presence of concurrent IDH1 or IDH2 mutations can significantly impact risk stratification, leading to an alteration in the prognostic profile. Several studies highlighted the adverse impact of IDH mutations on clinical outcomes in NPM1mut AML, indicating a poorer prognosis compared to NPM1mut cases without IDH mutations [18,19]. Indeed, the impact of IDH mutations on the clinical outcome of NPM1-mut AML is reflected in a higher rate of refractory disease, increased likelihood of relapse, and decreased response to standard chemotherapy regimens [18,19]. Additionally, the presence of IDH mutations in NPM1mut AML has significant implications for treatment strategies. Targeted therapies specifically designed to inhibit mutant IDH enzymes, such as ivosidenib (IDH1 inhibitor) and enasidenib (IDH2 inhibitor), have emerged as promising therapeutic options. These inhibitors aim to disrupt the abnormal metabolic processes associated with IDH mutations and have shown efficacy in inducing clinical responses, making them valuable additions to the therapeutic strategy for NPM1mut AML patients with IDH mutations [20,21]. For all of these reasons, early identification of these mutations in NPM1mut AML patients could be useful, when these therapeutic options are included in approved therapy, to personalize therapeutic approaches and enhance patients’ overall outcomes.

Canonical cancer-associated Ras mutations at codons G12, G13, or Q61 severely compromise the hydrolysis of GTP to GDP, leading to the accumulation of Ras-GTP and hyperactivation of Ras downstream signaling pathways. Mutations in the KRAS and NRAS genes are frequently observed in myeloid disorders (15–60%), including AML [22]. In our analysis, we detected some RAS mutations; however, the KRAS mutations identified had low allelic frequency and were not confirmed by RNAseq analysis. In contrast, we observed two mutations in the NRAS gene in two distinct patients at the RNA level (p.G13R; p.G12D). Of these, only the p.G13R mutation was present in the IonTorrent amplicon panel and was further confirmed by RNAseq analysis (NRAS p.G13R (NM_002524)). NRAS G12 and G13 mutations affect the intrinsic GTPase activity of NRAS by hindering the proper position of the Ras-GAP arginine finger within the catalytic site. In human cancers with NRAS mutations, G12 mutations are notably prevalent in myeloid leukemia.

By broadening the scope of our analysis to include less-expressed transcripts, we identified some polymorphisms associated with leukemia that could provide a starting point for further studies, focused on how these molecular changes can alter the evolution/progression of leukemia. For instance, we identified three patients with non-canonical variations in the DNMT3A genes, namely the R730C and R730H mutations, which result in amino acid changes at position 730 in the DNMT3A protein. These mutations have the potential to influence the function of DNMT3A and, consequently, the regulation of DNA methylation, a critical process in normal cellular functions. While these mutations have been previously identified in AML patients [23,24], the exact effects and clinical implications of these variations are currently unknown. While our primary focus is on mutations that can cause amino acid alterations, particularly those with established associations with hematological tumors, the RNAseq analysis permits us also to explore the genetic stability of transcription. RNAseq gives us additional information because a mutation identified at the RNA level may be translated into a protein with altered function; on the other hand, mutations found at the DNA level may be silent. However, at present, our tools do not enable us to understand whether the observed transcript alterations actually will be reflected at the protein level, thereby altering corresponding molecular pathways, or if they may be due to bioinformatic misinterpretations. To reduce these errors, several groups have worked to increase the performance of fusion analysis by integrating different algorithms to obtain a more reliable result [25,26,27]. Of note, some of the identified loci could play a role in the pathogenesis or progression of leukemia. The gene FAM65B is repeatedly shown as differentially methylated in some studies, and the CpG sites from chromosome 6 annotated to gene FAM65B had more variability in acute myeloid leukemia cases than in controls, suggesting its importance for lymphatic–hematopoietic cancers [28,29]. The function of FAM65B is to regulate the proliferation of transformed primary T cells; its forced expression blocks T cell mitosis, leading to G2 cell cycle arrest and apoptosis, while its absence increases clonal proliferation [30]. Intriguingly, the over-represented transcript alteration in our patients involved a genetic fusion between the first exon on FAB65B and RP11-367G6.3, a long noncoding RNA that shares the first exon with the FAM65B gene (Supplementary Figure S2). It is reasonable to hypothesize that those frame alterations, resulting from an incorrect splicing mechanism in this region, could affect the expression of FAM65B protein, favoring the leukemic clone with low expression of this gene. Our data and the existing literature suggest investigating the future FAM65B expression in AML patients and understanding how it can influence the course of leukemia.

This study aimed to identify potential genetic alterations, including low-frequency variations and clonal subtypes, through NGS transcript analysis within the specific cohort of NPM1mut AML. Our findings highlight the need for accurate genetic analyses that can improve patient characterization. This approach can extend the scope of analysis to encompass low-penetrance variations in the population, currently excluded from myeloid panels, but potentially related to clone selection and treatment response. Furthermore, it allows a comprehensive evaluation of the real expression of exome transcripts in leukemic clones. In the future, thanks to cost reduction and advancements in data analysis, this approach will be increasingly competitive. However, some challenges need to be overcome. It is crucial to improve bioinformatic knowledge to increase the reliability of NGS analyses and to overcome problems related to errors in identifying insertions and deletions. It is necessary to improve the procedures to obtain a good compromise between the severity of the analysis and the real presence of specific variations in particular areas. RNA sequencing has the potential to simplify certain situations by revealing the transcripts expressed by the various tumor clones at a specific moment. However, challenges arise in defining and selecting the appropriate number of transcripts for which the called variation is valid. At the same time, it is necessary to work at the experimental level to understand the biological/clinical significance of the identified alterations, whether they involve amino acid changes or genetic fusions. This understanding is crucial to make these findings usable for future personalized leukemic therapies.

In summary, our study provides a comprehensive overview of concomitant mutations within our NPM1-mutated leukemia cohort, highlighting significant differences between patient fingerprinting at the genetic/transcriptional level and “standard” diagnostic assessment.

The advantage of using RNAseq compared to classical molecular biology methods and also compared to panel-based NGS analyses is twofold.

In the short term, RNAseq enables the optimization of a plethora of mutations and chromosomal rearrangements/aberrations defined by the guidelines. Currently, these assessments rely on different molecular methodologies, often lacking generalizability and standardization across laboratories. The evaluation of the transcripts effectively produced by the patient’s leukemic cells will allow us to understand what promotes the genesis of the disease and/or how resistance to therapy can evolve at the transcript level. Furthermore, analysis at the RNA level can reduce the complexity of genetic evaluation by excluding non-expressed GVs that do not contribute to the phenotype. By doing so, it is possible to identify any clones with mutations in key genes that could, if identified early, change the course of the disease, and optimize the patient’s therapy.

In the long term, an exomic RNA level approach could bring further advantages. In our analysis, which evaluated the feasibility and convenience of this approach, we focused on the variations already identified as related to AML to confirm the validity of our strategy. Unfortunately, the small cohort of NPM1mut FLT3wt patients did not allow us to conduct a sufficiently robust statistical analysis to dissect the different genetic variations we have identified as cancer-related. In addition to this inconvenience, the heterogeneity of AML patients, who are very complex and fragmented at a clinical, genetic, and molecular level, makes the statistical analysis even more challenging. Expanding the exomic approach, with standardized methods, to more laboratories dealing with AML, we could overcome this problem by providing a clearer picture of currently uninteresting chromosomal regions or genes that may have future significance. Especially in a context where leukemias and tumors stop being organ diseases and become molecular diseases, also drugs developed in other molecular contexts could be evaluated for patients with those specific defects.

The apparent simplification of the complex molecular landscape of patients with NPM1-mutated AML causes a potential underestimation of risk within this particular cohort. The imperative for accurately directing patients to the optimal therapy necessitates genetic evaluation utilizing innovative technologies, such as NGS. Employing these advanced approaches is crucial for identifying the risk profile of each patient and tailoring the appropriate personalized therapeutic strategies accordingly.

## 4. Materials and Methods

### 4.1. Sample Cohort

We carried out a retrospective study utilizing diagnostic material (RNA and/or DNA) extracted from AML patients. We enrolled individuals aged between 18 and 90 years, with no gender preference, presenting with mutated NPM1, a normal karyotype, and classified as wild type for other common myeloid-associated mutations at diagnosis. Diagnostic analysis included screening for NPM1, IDH1, IDH2, FLT3-ITD, and FLT3-TKD variants following international guidelines [4]. All patients were stratified with a favorable prognosis according to clinical guidelines.

We performed RNAseq analysis on diagnostic material from 19 patients, and DNAseq analysis from 17 patients. Thirteen patients were evaluated with both NGS technologies, while the RNA sample from one patient failed the library quality controls and was excluded from analysis. All specimens were collected at the University of Turin, Internal Medicine-San Luigi Gonzaga Hospital (Orbassano, Turin, Italy). The study was conducted in accordance with the Declaration of Helsinki, and approved by the Ethics Committee of AOU San Luigi Gonzaga (protocol number 201/2014). Written informed consent was obtained from each enrolled subject.

### 4.2. NGS Analysis

For DNA analysis, we generated NGS libraries using Ion Torrent™ Oncomine™ Pan-Cancer and IonTorrent System (Thermo Fisher Scientific, Waltham, MA, USA) following the manufacturer’s procedures. This panel not only identifies mutations associated with the myeloid lineage but, on a broader scale, encompasses all variations in genes previously correlated with tumors. Among these, single nucleotide variations (SNVs) and small insertions/deletions were identified in the “hotspot regions” of the following genes: AKT1, ALK, AR, ARAF, BRAF, CHEK2, CTNNB1, DDR2, EGFR, ERBB2, ERBB3, ESR1, FGFR1, FGFR2, FGFR3, FGFR4, FLT3, GNA11, GNAQ, GNAS, HRAS, IDH1, IDH2, KIT, KRAS, MAP2K1, MAP2K2, MET, MTOR, NRAS, NTRK1, NTRK3, PDGFRA, PIK3CA, RAF1, RET, ROS1, SF3B1, SMAD4, SMO, APC, FBXW7, PTEN, and TP53. Subsequently, to maximize the identification of pathological mutations, we applied a rigorous and widely validated bioinformatic pipeline. In particular, the files obtained from the sequencer were aligned to the human genome using the BWA-mem software and the GRCh38 genome release. The generated files were indexed, sorted, and marked to exclude duplicate sequences with Samtools/Picard. To identify genetic variants, we used the “GATK best practices” [31]. Variants that passed the filters were subsequently annotated using a Perl script that uses Annovar databases [32].

For RNA sequencing, we generated the libraries starting from patient RNA, using the Illumina TruSeq Stranded Total RNA Library Prep Gold and Novaseq 6000 System (Illumina, San Diego, CA, USA), following the Illumina standard procedure and kits. To evaluate the sequences obtained, we performed a two-step alignment to the genome (GRCh38 genome release) using the STAR software. The resulting file was processed to eliminate artefactual duplicates, and the sequence of each transcript was divided into the exons corresponding to the respective gene on the reference genome (Hg38), using the “SplitNCigarReads” algorithm. The calling of genetic variants compared to the reference genome was performed with the Mutect2 software of the Genome Analysis Toolkit (GATK) version 4.1.8.1 package (https://gatk.broadinstitute.org, accessed on 13 January 2024). Briefly, Mutect2 was run in “tumor only” mode following “GATK best practices”. To filter potential germline variants, we used a “panel of normals” and a population reference of germline variants, generated by the 1000 Genomes Project (1000GP) and the Genome Aggregation Database (gnomAD), respectively. Following mutation calling, putative germline SNVs and any INDELs were filtered using FilterMutectCalls. Variants that passed the filters were subsequently annotated using a Perl script that uses Annovar databases.

Chromosomal fusions were predicted using STAR-Fusion (v1.9) [33]. First, the STAR (v2.7) aligner was used to align RNA-seq reads from each sample to the human genome. The resulting Chimeric.out.junction files, generated by STAR and containing candidate chimeric reads, were then analyzed by STAR-Fusion standard parameters as indicated on the wiki page (https://github.com/STAR-Fusion/STAR-Fusion/wiki, accessed on 22 January 2024), with reference to the CTAT genome library GRCh38_gencode_v22_CTAT_lib.

## Figures and Tables

**Table 1:** Non-synonymous genetic variations identified by total RNA sequencing analysis.

Chr Pos Ref Alt	Representative Amino Acid Change (Transcript)	dbSNP	Cancer (Sequencing Depth > 25)	Leukemia (Sequencing Depth > 25)	Cancer (Sequencing Depth > 100)	Leukemia (Sequencing Depth > 100)
11 108304736 A T	ATM p.D1853V (NM_000051)	rs1801673	2			
11 108317431 A T	ATM p.Y2086F (NM_000051)	rs730881380	1		1	
11 108331520 T C	ATM p.M2531T (NM_000051)	rs587781365	1			
20 56386485 A T	AURKA p.F31I (NM_003600)	rs2273535	2			
11 119284996 A G	CBL p.M487V (NM_005188)	rs17848896	1	1		
12 57750985 C G	CDK4 p.V154L (NM_000075)	rs563692823	1			
12 12717864 G T	CDKN1B p.G9W (NM_004064)	.	1		1	
14 95099907 C T	DICER1 p.G1360E (NM_001195573)	.	1			
2 25234374 G A	DNMT3A p.R882H (NM_175629)	rs377577594	2	2		
2 25234373 C T	DNMT3A p.R882H (NM_175629)	rs147001633	1	1		
13 28018492 T C	FLT3 p.D839G (NM_004119)	rs991132188	1	1		
11 67585218 A G	GSTP1 p.I105V (NM_000852)	rs1695	9		9	
15 90088702 C T	IDH2 p.R140Q (NM_002168)	rs121913502	1	1	1	1
2 47410166 G A	MSH2 p.V81I (NM_001258281)	rs773125415	1		1	
1 11796321 G A	MTHFR p.A263V (NM_001330358)	rs1801133	9		1	
5 7870860 A G	MTRR p.I22M (NM_001364440)	rs1801394	11			
1 45332190 C A	MUTYH p.E275D (NM_001048174)	rs863224700	1			
16 69711242 G A	NQO1 p.P187S (NM_000903)	rs1800566	1	1		
1 114716126 C T	NRAS p.G12D (NM_002524)	rs121913237	1	1	1	1
1 114716124 C G	NRAS p.G13R (NM_002524)	rs121434595	1	1		
7 5995556 C A	PMS2 pR294L (NM_001322006)	.	1			
19 50399016 G C	POLD1 p.E55D (NM_001308632)	.	1			
19 50402013 C T	POLD1 p.E55D (NM_001308632)	rs144770820	1		1	
15 90970444 T C	PRC1 pY511C (NM_003981)	rs12911192	1			
12 112450395 C T	PTPN11 p.A72V (NM_001330437)	rs121918454	1	1	1	1
12 112450394 G A	PTPN11 p.A72T (NM_001330437)	rs121918453	1	1	1	1
12 112450406 G A	PTPN11 p.E76K (NM_001330437)	rs121918464	1	1	1	1
11 48123823 A C	PTPRJ p.Q276P (NM_001098503)	rs1566734	5		2	
5 132609190 G A	RAD50 p.G968E (NM_005732)	rs199895166	1			
1 182585422 C T	RNASEL p.R462Q (NM_021133)	rs486907	6			
17 18328782 G A	SHMT1 p.L474F (NM_004169)	rs1979277	2			
21 45537880 T C	SLC19A1 p.H27R (NM_001205206)	rs1051266	10			
17 7676154 G C	TP53 p.P72R (NM_000546)	rs1042522	14		5	
6 96552610 C G	UFL1 p.P705R (NM_015323)	rs199880163	1			

**Table 2:** Chromosomal rearrangements identified in fusion analysis.

Chr Fusion	Count	Splice Junctions	Type
C22orf39--IGH@-ext	1	INCL_NON_REF_SPLICE	INTERCHROMOSOMAL[chr22--chr14]
CTC-786C10.1--RP11-680G10.1	1	ONLY_REF_SPLICE	INTRACHROMOSOMAL[chr16:0.17Mb]
HIRA--IGH@-ext	2	INCL_NON_REF_SPLICE	INTERCHROMOSOMAL[chr22--chr14]
LILRB1--AC010518.2	2	ONLY_REF_SPLICE	INTRACHROMOSOMAL[chr19:0.32Mb]
PFKFB3--RP11-563J2.2	2	ONLY_REF_SPLICE	INTRACHROMOSOMAL[chr10:0.04Mb];NEIGHBORS[42142]
RP11-367G6.3--FAM65B	7	ONLY_REF_SPLICE	INTRACHROMOSOMAL[chr6:0.08Mb];NEIGHBORS[78900]
RP11-444D3.1--SOX5	1	ONLY_REF_SPLICE	[“SOX5:Oncogene”];INTRACHROMOSOMAL[chr12:0.26Mb]
RP11-632K20.2--RP11-632K20.7	4	INCL_NON_REF_SPLICE	INTRACHROMOSOMAL[chr15:0.02Mb];LOCAL_REARRANGEMENT:−:[19502]
RP1-90G24.6--RP1-90G24.10	1	ONLY_REF_SPLICE	INTRACHROMOSOMAL[chr22:0.00Mb];LOCAL_REARRANGEMENT:+:[3754]
SLC7A5P1--SMG1	2	INCL_NON_REF_SPLICE	INTRACHROMOSOMAL[chr16:10.69Mb]
ST3GAL1--NDRG1	1	ONLY_REF_SPLICE	[“NDRG1:Oncogene”];INTRACHROMOSOMAL[chr8:0.15Mb]

**Table 3:** Summary of genetic alterations identified in our analysis.

# ID ^¥^	DNAseq > 100 (NM_) (AF%)	RNAseq Sequencing Depth > 25 (NM_) (TR%) *	Chr Fusion
R12			RP11-367G6.3--FAM65B
R13		**PTPN11 p.A72V (NM_001330437) (24%)**, NQO1 p.P187S (NM_000903) (52%)%), CEBPA p.H59Afs*84 (NM_001287424) (58%)	SLC7A5P1--SMG1; RP11-367G6.3--FAM65B
R14/D1	NRAS p.G13R (NM_002524) (34%)	NRAS p.G13R (NM_002524) (20%); DNMT3A p.R882H (NM_175629) (47%)	ST3GAL1--NDRG1; SLC7A5P1--SMG1; PFKFB3--RP11-563J2.2
R15/D2	KRAS (NM_033360) p.Q61H (6%)	DNMT3A p.R882H (NM_175629) (25%)	No fusion
R16		**IDH2 p.R140Q (NM_002168) (36%)**	RP11-632K20.2--RP11-632K20.7
R17			RP11-367G6.3--FAM65B
R18			RP11-632K20.2--RP11-632K20.7
R20			RP11-632K20.2--RP11-632K20.7
R21		**PTPN11 p.A72T (NM_001330437) (24%),** FLT3 p.D839G (NM_004119) (11%);	RP11-367G6.3--FAM65B; RP1-90G24.6--RP1-90G24.10
R24			RP11-367G6.3--FAM65B
R25			LILRB1--AC010518.2
R26/D5	KRAS p.G13D (NM_033360) (12%)	CBL p.M487V (NM_005188) (39%)	RP11-444D3.1--SOX5; PFKFB3--RP11-563J2.2; CTC-786C10.1--RP11-680G10.1
R5		**NRAS p.G12D (NM_002524) (48%),** DNMT3A p.R882H (NM_175629) (36%)	No fusion
R6		NBN p.S53Cfs*9 (NM_002485) (2%)	RP11-367G6.3--FAM65B; HIRA--IGH@-ext
R7		**PTPN11 p.E76K (NM_001330437) (48%)**	LILRB1--AC010518.2
R9			RP11-367G6.3--FAM65B; C22orf39--IGH@-ext; RP11-632K20.2--RP11-632K20.7
D4	IDH2 p.R140Q (NM_002168) (6%)	No RNA	No RNA

^¥^ R: RNA ID; D: DNA ID. * In bold, the GVs identified also with sequencing depth > 100 transcripts in RNA analysis.

## Data Availability

Dataset available on request from the authors.

## Supplementary Materials

The following supporting information can be downloaded at: https://www.mdpi.com/article/10.3390/ijms25073631/s1.
