# Supplementary material for: Comprehensive Molecular Profiling of NPM1-Mutated Acute Myeloid Leukemia Using RNAseq Approach
Source: Int J Mol Sci. 2024 Mar 24;25(7):3631. doi: 10.3390/ijms25073631 (PMC11011776; doi:10.3390/ijms25073631)
Supplement: Supplementary file 1 [file ijms-25-03631-s001.zip › Supplementary.Figures.pdf]

Supplementary material

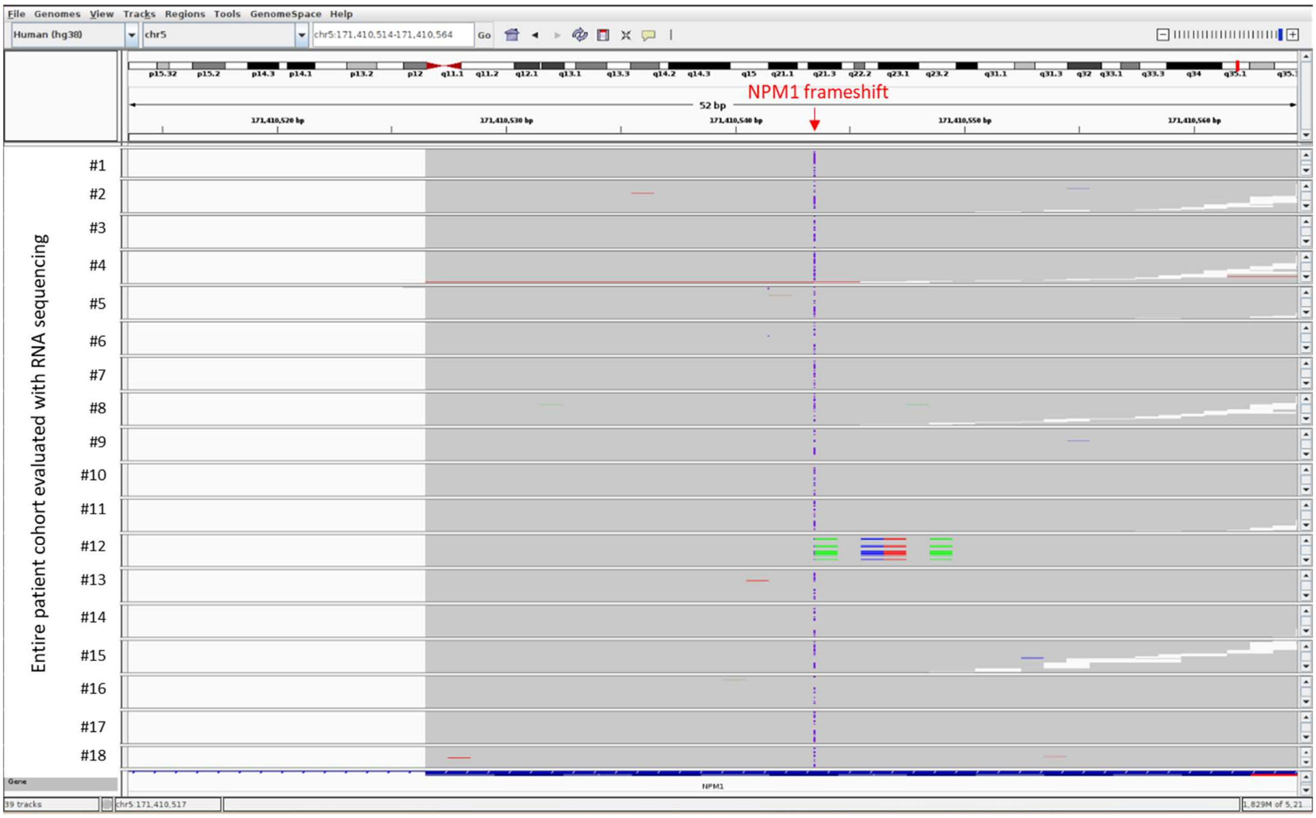

Supplementary Figure S1. NPM1 genomic alignment. We observed insertions in all patients but the bioinformatic algorithm called it only 6 times. Red arrow indicated the insertion, in purple in each patient.

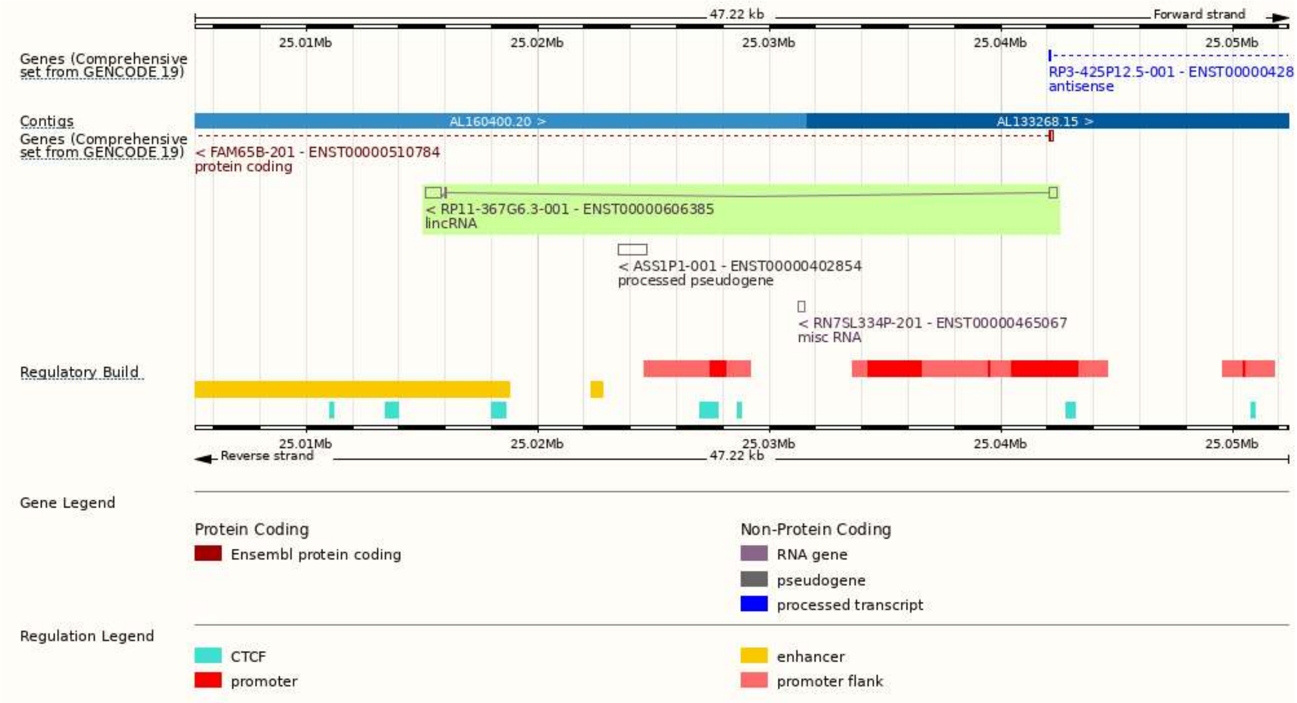

Supplementary Figure S2. Locus FAM65B gene. The over-represented transcript alteration in our patients involved a genetic fusion between the first exon on FAB65B and RP11-367G6.3, a long noncoding RNA (green box) that shares the first exon with the FAM65B gene (indicated in garnet).
